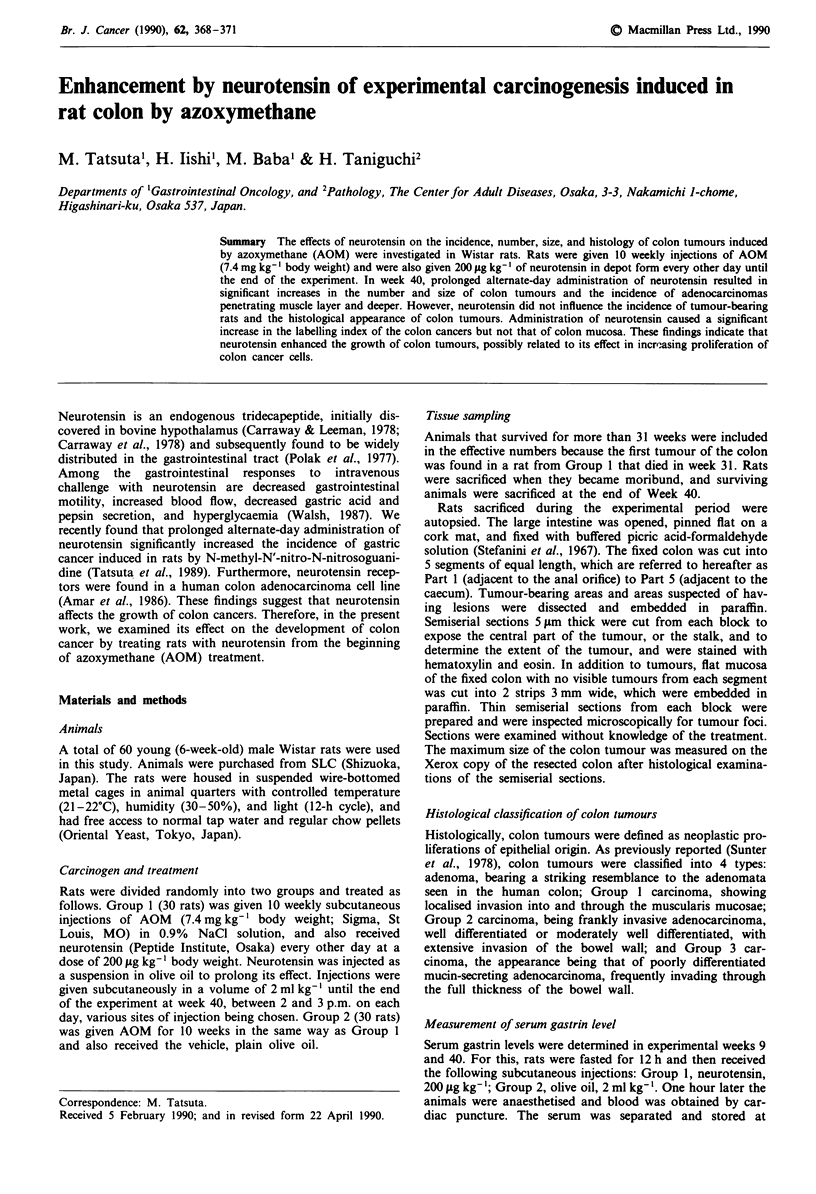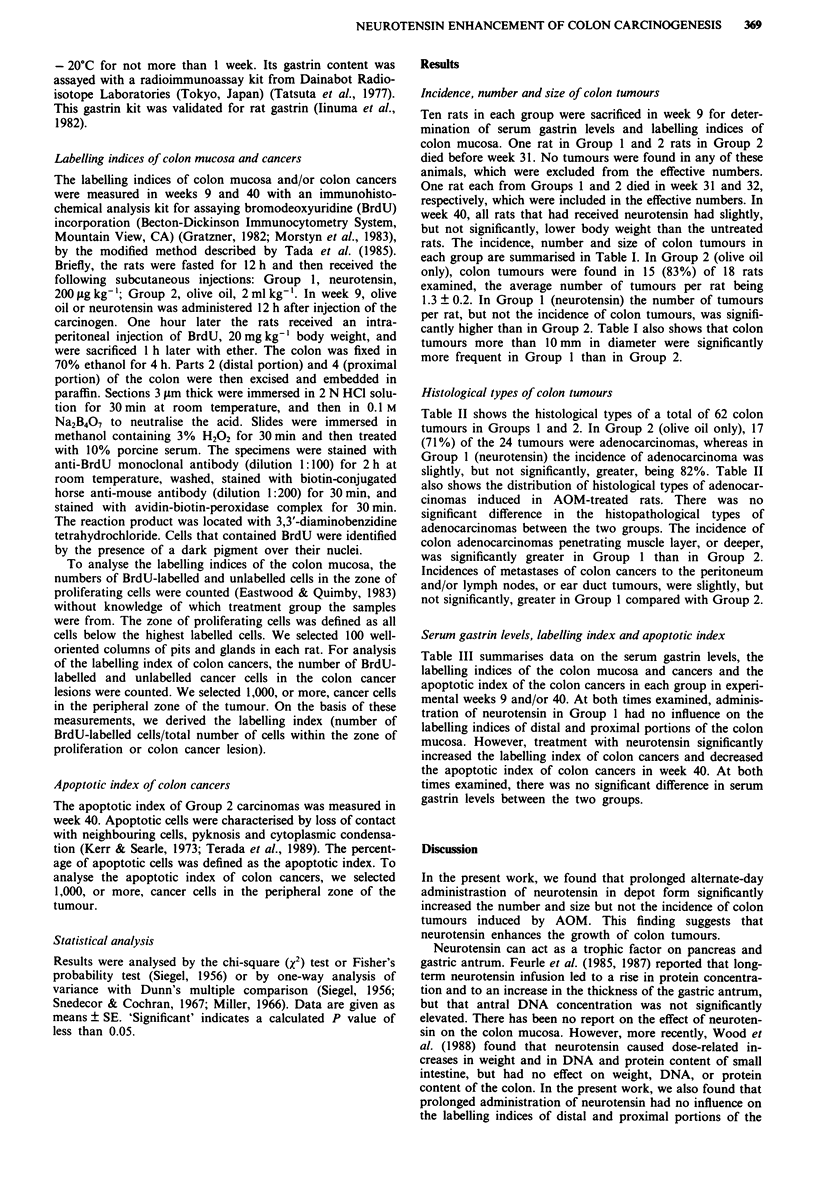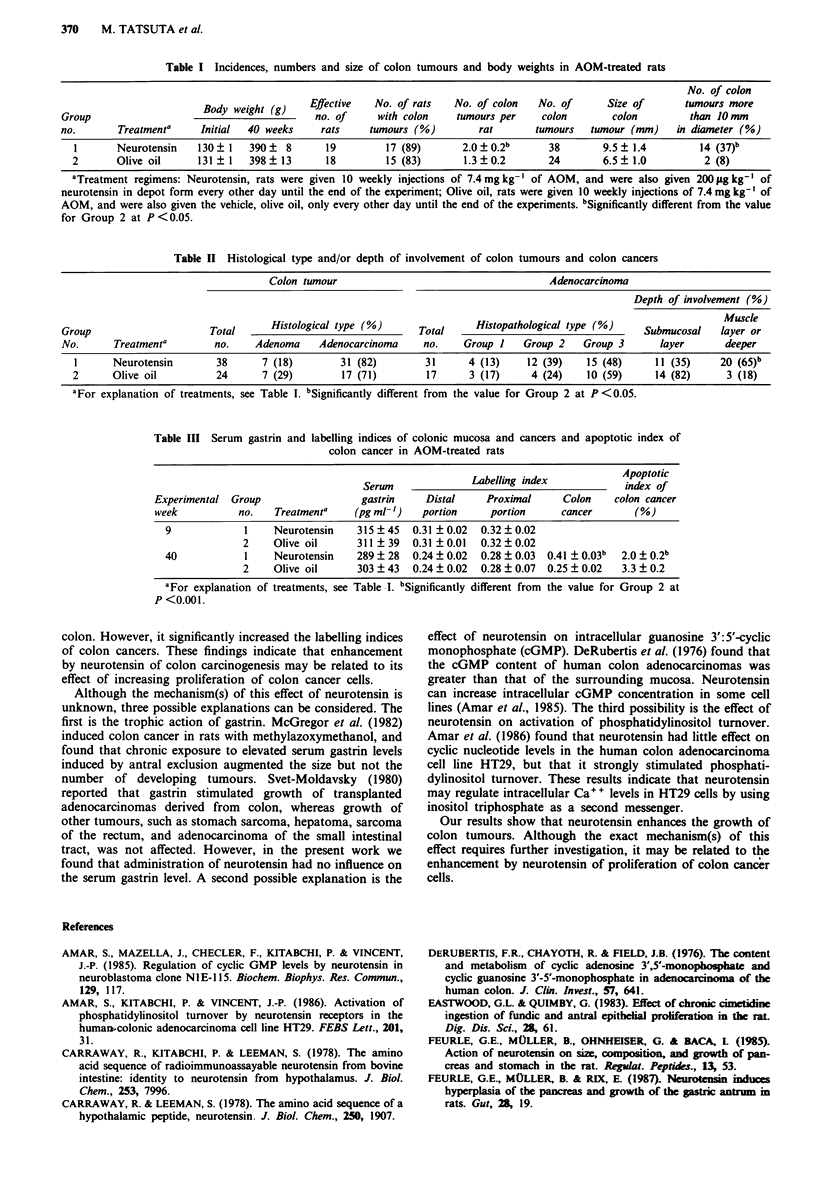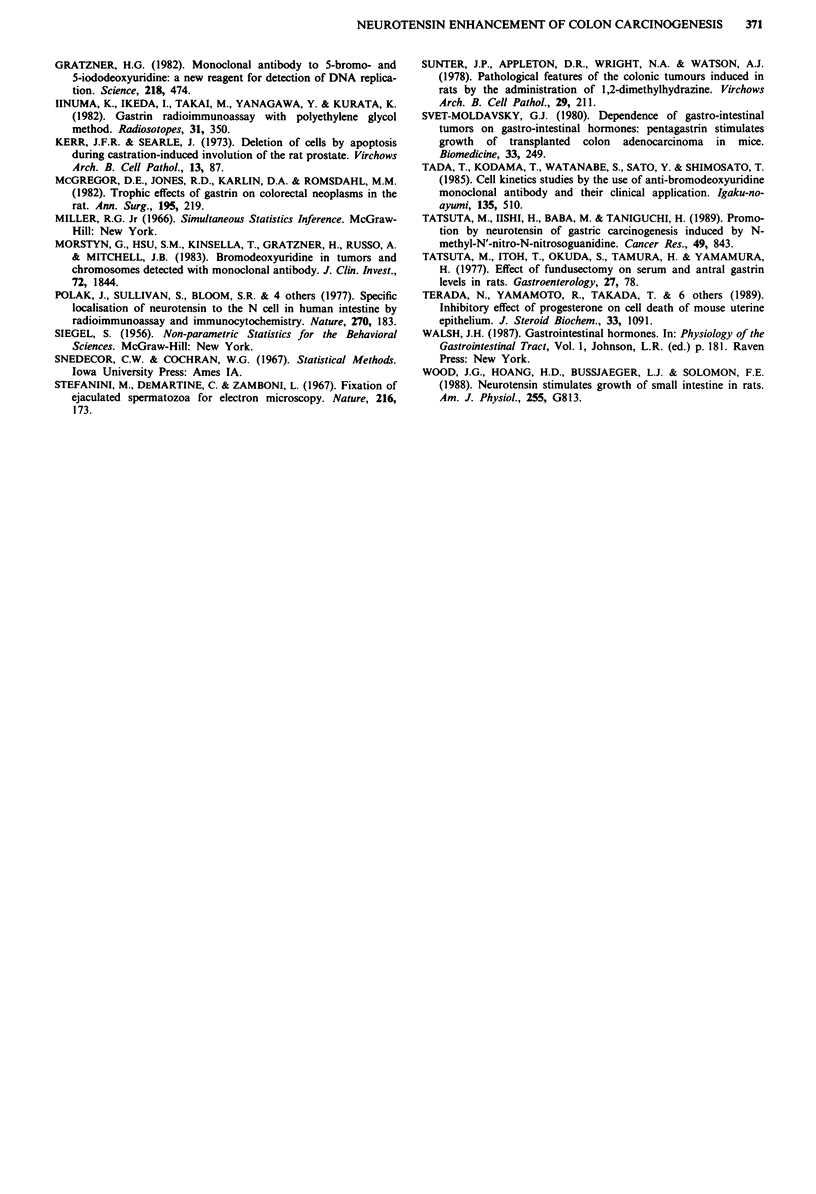# Enhancement by neurotensin of experimental carcinogenesis induced in rat colon by azoxymethane.

**DOI:** 10.1038/bjc.1990.299

**Published:** 1990-09

**Authors:** M. Tasuta, H. Iishi, M. Baba, H. Taniguchi

**Affiliations:** Department of Gastrointestinal Oncology, The Center for Adult Diseases, Osaka, Japan.

## Abstract

The effects of neurotensin on the incidence, number, size, and histology of colon tumours induced by azoxymethane (AOM) were investigated in Wistar rats. Rats were given 10 weekly injections of AOM (7.4 mg kg-1 body weight) and were also given 200 micrograms kg-1 of neurotensin in depot form every other day until the end of the experiment. In week 40, prolonged alternate-day administration of neurotensin resulted in significant increases in the number and size of colon tumours and the incidence of adenocarcinomas penetrating muscle layer and deeper. However, neurotensin did not influence the incidence of tumour-bearing rats and the histological appearance of colon tumours. Administration of neurotensin caused a significant increase in the labelling index of the colon cancers but not that of colon mucosa. These findings indicate that neurotensin enhanced the growth of colon tumours, possibly related to its effect in increasing proliferation of colon cancer cells.


Br. J. Cancer (1990), 62, 368-371 © Macmillan Press Ltd., 1990

### Enhancement by neurotensin of experimental carcinogenesis induced in rat colon by azoxymethane

M. Tatsuta[1], H. Iishi[1], M. Baba[1] & H. Taniguchi[2]

*Departments of [1]Gastrointestinal Oncology, and [2]Pathology, The Center for Adult Diseases, Osaka, 3-3, Nakamichi 1-chome, Higashinari-ku, Osaka 537, Japan.*

**Summary** The effects of neurotensin on the incidence, number, size, and histology of colon tumours induced by azoxymethane (AOM) were investigated in Wistar rats. Rats were given 10 weekly injections of AOM (7.4 mg $kg^{-1}$ body weight) and were also given 200 $\mu g\ kg^{-1}$ of neurotensin in depot form every other day until the end of the experiment. In week 40, prolonged alternate-day administration of neurotensin resulted in significant increases in the number and size of colon tumours and the incidence of adenocarcinomas penetrating muscle layer and deeper. However, neurotensin did not influence the incidence of tumour-bearing rats and the histological appearance of colon tumours. Administration of neurotensin caused a significant increase in the labelling index of the colon cancers but not that of colon mucosa. These findings indicate that neurotensin enhanced the growth of colon tumours, possibly related to its effect in increasing proliferation of colon cancer cells.

Neurotensin is an endogenous tridecapeptide, initially discovered in bovine hypothalamus (Carraway & Leeman, 1978; Carraway *et al.*, 1978) and subsequently found to be widely distributed in the gastrointestinal tract (Polak *et al.*, 1977). Among the gastrointestinal responses to intravenous challenge with neurotensin are decreased gastrointestinal motility, increased blood flow, decreased gastric acid and pepsin secretion, and hyperglycaemia (Walsh, 1987). We recently found that prolonged alternate-day administration of neurotensin significantly increased the incidence of gastric cancer induced in rats by N-methyl-N′-nitro-N-nitrosoguanidine (Tatsuta *et al.*, 1989). Furthermore, neurotensin receptors were found in a human colon adenocarcinoma cell line (Amar *et al.*, 1986). These findings suggest that neurotensin affects the growth of colon cancers. Therefore, in the present work, we examined its effect on the development of colon cancer by treating rats with neurotensin from the beginning of azoxymethane (AOM) treatment.

### Materials and methods

### *Animals*

A total of 60 young (6-week-old) male Wistar rats were used in this study. Animals were purchased from SLC (Shizuoka, Japan). The rats were housed in suspended wire-bottomed metal cages in animal quarters with controlled temperature (21–22°C), humidity (30–50%), and light (12-h cycle), and had free access to normal tap water and regular chow pellets (Oriental Yeast, Tokyo, Japan).

### *Carcinogen and treatment*

Rats were divided randomly into two groups and treated as follows. Group 1 (30 rats) was given 10 weekly subcutaneous injections of AOM (7.4 mg $kg^{-1}$ body weight; Sigma, St Louis, MO) in 0.9% NaCl solution, and also received neurotensin (Peptide Institute, Osaka) every other day at a dose of 200 $\mu g\ kg^{-1}$ body weight. Neurotensin was injected as a suspension in olive oil to prolong its effect. Injections were given subcutaneously in a volume of 2 ml $kg^{-1}$ until the end of the experiment at week 40, between 2 and 3 p.m. on each day, various sites of injection being chosen. Group 2 (30 rats) was given AOM for 10 weeks in the same way as Group 1 and also received the vehicle, plain olive oil.

Correspondence: M. Tatsuta.
Received 5 February 1990; and in revised form 22 April 1990.

### *Tissue sampling*

Animals that survived for more than 31 weeks were included in the effective numbers because the first tumour of the colon was found in a rat from Group 1 that died in week 31. Rats were sacrificed when they became moribund, and surviving animals were sacrificed at the end of Week 40.

Rats sacrificed during the experimental period were autopsied. The large intestine was opened, pinned flat on a cork mat, and fixed with buffered picric acid-formaldehyde solution (Stefanini *et al.*, 1967). The fixed colon was cut into 5 segments of equal length, which are referred to hereafter as Part 1 (adjacent to the anal orifice) to Part 5 (adjacent to the caecum). Tumour-bearing areas and areas suspected of having lesions were dissected and embedded in paraffin. Semiserial sections 5 µm thick were cut from each block to expose the central part of the tumour, or the stalk, and to determine the extent of the tumour, and were stained with hematoxylin and eosin. In addition to tumours, flat mucosa of the fixed colon with no visible tumours from each segment was cut into 2 strips 3 mm wide, which were embedded in paraffin. Thin semiserial sections from each block were prepared and were inspected microscopically for tumour foci. Sections were examined without knowledge of the treatment. The maximum size of the colon tumour was measured on the Xerox copy of the resected colon after histological examinations of the semiserial sections.

### *Histological classification of colon tumours*

Histologically, colon tumours were defined as neoplastic proliferations of epithelial origin. As previously reported (Sunter *et al.*, 1978), colon tumours were classified into 4 types: adenoma, bearing a striking resemblance to the adenomata seen in the human colon; Group 1 carcinoma, showing localised invasion into and through the muscularis mucosae; Group 2 carcinoma, being frankly invasive adenocarcinoma, well differentiated or moderately well differentiated, with extensive invasion of the bowel wall; and Group 3 carcinoma, the appearance being that of poorly differentiated mucin-secreting adenocarcinoma, frequently invading through the full thickness of the bowel wall.

### *Measurement of serum gastrin level*

Serum gastrin levels were determined in experimental weeks 9 and 40. For this, rats were fasted for 12 h and then received the following subcutaneous injections: Group 1, neurotensin, 200 $\mu g\ kg^{-1}$; Group 2, olive oil, 2 ml $kg^{-1}$. One hour later the animals were anaesthetised and blood was obtained by cardiac puncture. The serum was separated and stored at

− 20°C for not more than 1 week. Its gastrin content was assayed with a radioimmunoassay kit from Dainabot Radioisotope Laboratories (Tokyo, Japan) (Tatsuta *et al.*, 1977). This gastrin kit was validated for rat gastrin (Iinuma *et al.*, 1982).

*Labelling indices of colon mucosa and cancers*

The labelling indices of colon mucosa and/or colon cancers were measured in weeks 9 and 40 with an immunohistochemical analysis kit for assaying bromodeoxyuridine (BrdU) incorporation (Becton-Dickinson Immunocytometry System, Mountain View, CA) (Gratzner, 1982; Morstyn *et al.*, 1983), by the modified method described by Tada *et al.* (1985). Briefly, the rats were fasted for 12 h and then received the following subcutaneous injections: Group 1, neurotensin, 200 $\mu g\ kg^{-1}$; Group 2, olive oil, 2 $ml\ kg^{-1}$. In week 9, olive oil or neurotensin was administered 12 h after injection of the carcinogen. One hour later the rats received an intraperitoneal injection of BrdU, 20 $mg\ kg^{-1}$ body weight, and were sacrificed 1 h later with ether. The colon was fixed in 70% ethanol for 4 h. Parts 2 (distal portion) and 4 (proximal portion) of the colon were then excised and embedded in paraffin. Sections 3 $\mu m$ thick were immersed in 2 N HCl solution for 30 min at room temperature, and then in 0.1 M $Na_2B_4O_7$ to neutralise the acid. Slides were immersed in methanol containing 3% $H_2O_2$ for 30 min and then treated with 10% porcine serum. The specimens were stained with anti-BrdU monoclonal antibody (dilution 1:100) for 2 h at room temperature, washed, stained with biotin-conjugated horse anti-mouse antibody (dilution 1:200) for 30 min, and stained with avidin-biotin-peroxidase complex for 30 min. The reaction product was located with 3,3′-diaminobenzidine tetrahydrochloride. Cells that contained BrdU were identified by the presence of a dark pigment over their nuclei.

To analyse the labelling indices of the colon mucosa, the numbers of BrdU-labelled and unlabelled cells in the zone of proliferating cells were counted (Eastwood & Quimby, 1983) without knowledge of which treatment group the samples were from. The zone of proliferating cells was defined as all cells below the highest labelled cells. We selected 100 well-oriented columns of pits and glands in each rat. For analysis of the labelling index of colon cancers, the number of BrdU-labelled and unlabelled cancer cells in the colon cancer lesions were counted. We selected 1,000, or more, cancer cells in the peripheral zone of the tumour. On the basis of these measurements, we derived the labelling index (number of BrdU-labelled cells/total number of cells within the zone of proliferation or colon cancer lesion).

*Apoptotic index of colon cancers*

The apoptotic index of Group 2 carcinomas was measured in week 40. Apoptotic cells were characterised by loss of contact with neighbouring cells, pyknosis and cytoplasmic condensation (Kerr & Searle, 1973; Terada *et al.*, 1989). The percentage of apoptotic cells was defined as the apoptotic index. To analyse the apoptotic index of colon cancers, we selected 1,000, or more, cancer cells in the peripheral zone of the tumour.

*Statistical analysis*

Results were analysed by the chi-square ($\chi^2$) test or Fisher's probability test (Siegel, 1956) or by one-way analysis of variance with Dunn's multiple comparison (Siegel, 1956; Snedecor & Cochran, 1967; Miller, 1966). Data are given as means ± SE. 'Significant' indicates a calculated $P$ value of less than 0.05.

### Results

*Incidence, number and size of colon tumours*

Ten rats in each group were sacrificed in week 9 for determination of serum gastrin levels and labelling indices of colon mucosa. One rat in Group 1 and 2 rats in Group 2 died before week 31. No tumours were found in any of these animals, which were excluded from the effective numbers. One rat each from Groups 1 and 2 died in week 31 and 32, respectively, which were included in the effective numbers. In week 40, all rats that had received neurotensin had slightly, but not significantly, lower body weight than the untreated rats. The incidence, number and size of colon tumours in each group are summarised in Table I. In Group 2 (olive oil only), colon tumours were found in 15 (83%) of 18 rats examined, the average number of tumours per rat being 1.3 ± 0.2. In Group 1 (neurotensin) the number of tumours per rat, but not the incidence of colon tumours, was significantly higher than in Group 2. Table I also shows that colon tumours more than 10 mm in diameter were significantly more frequent in Group 1 than in Group 2.

*Histological types of colon tumours*

Table II shows the histological types of a total of 62 colon tumours in Groups 1 and 2. In Group 2 (olive oil only), 17 (71%) of the 24 tumours were adenocarcinomas, whereas in Group 1 (neurotensin) the incidence of adenocarcinoma was slightly, but not significantly, greater, being 82%. Table II also shows the distribution of histological types of adenocarcinomas induced in AOM-treated rats. There was no significant difference in the histopathological types of adenocarcinomas between the two groups. The incidence of colon adenocarcinomas penetrating muscle layer, or deeper, was significantly greater in Group 1 than in Group 2. Incidences of metastases of colon cancers to the peritoneum and/or lymph nodes, or ear duct tumours, were slightly, but not significantly, greater in Group 1 compared with Group 2.

*Serum gastrin levels, labelling index and apoptotic index*

Table III summarises data on the serum gastrin levels, the labelling indices of the colon mucosa and cancers and the apoptotic index of the colon cancers in each group in experimental weeks 9 and/or 40. At both times examined, administration of neurotensin in Group 1 had no influence on the labelling indices of distal and proximal portions of the colon mucosa. However, treatment with neurotensin significantly increased the labelling index of colon cancers and decreased the apoptotic index of colon cancers in week 40. At both times examined, there was no significant difference in serum gastrin levels between the two groups.

### Discussion

In the present work, we found that prolonged alternate-day administrastion of neurotensin in depot form significantly increased the number and size but not the incidence of colon tumours induced by AOM. This finding suggests that neurotensin enhances the growth of colon tumours.

Neurotensin can act as a trophic factor on pancreas and gastric antrum. Feurle *et al.* (1985, 1987) reported that long-term neurotensin infusion led to a rise in protein concentration and to an increase in the thickness of the gastric antrum, but that antral DNA concentration was not significantly elevated. There has been no report on the effect of neurotensin on the colon mucosa. However, more recently, Wood *et al.* (1988) found that neurotensin caused dose-related increases in weight and in DNA and protein content of small intestine, but had no effect on weight, DNA, or protein content of the colon. In the present work, we also found that prolonged administration of neurotensin had no influence on the labelling indices of distal and proximal portions of the

**Table I** Incidences, numbers and size of colon tumours and body weights in AOM-treated rats

| Group no. | Treatment[a] | Body weight (g) Initial | 40 weeks | Effective no. of rats | No. of rats with colon tumours (%) | No. of colon tumours per rat | No. of colon tumours | Size of colon tumour (mm) | No. of colon tumours more than 10 mm in diameter (%) |
|---|---|---|---|---|---|---|---|---|---|
| 1 | Neurotensin | 130 ± 1 | 390 ± 8 | 19 | 17 (89) | 2.0 ± 0.2[b] | 38 | 9.5 ± 1.4 | 14 (37)[b] |
| 2 | Olive oil | 131 ± 1 | 398 ± 13 | 18 | 15 (83) | 1.3 ± 0.2 | 24 | 6.5 ± 1.0 | 2 (8) |

[a]Treatment regimens: Neurotensin, rats were given 10 weekly injections of 7.4 mg $kg^{-1}$ of AOM, and were also given 200 µg $kg^{-1}$ of neurotensin in depot form every other day until the end of the experiment; Olive oil, rats were given 10 weekly injections of 7.4 mg $kg^{-1}$ of AOM, and were also given the vehicle, olive oil, only every other day until the end of the experiments. [b]Significantly different from the value for Group 2 at $P < 0.05$.

**Table II** Histological type and/or depth of involvement of colon tumours and colon cancers

| Group No. | Treatment[a] | Colon tumour: Total no. | Histological type (%): Adenoma | Adenocarcinoma | Adenocarcinoma: Total no. | Histopathological type (%): Group 1 | Group 2 | Group 3 | Depth of involvement (%): Submucosal layer | Muscle layer or deeper |
|---|---|---|---|---|---|---|---|---|---|---|
| 1 | Neurotensin | 38 | 7 (18) | 31 (82) | 31 | 4 (13) | 12 (39) | 15 (48) | 11 (35) | 20 (65)[b] |
| 2 | Olive oil | 24 | 7 (29) | 17 (71) | 17 | 3 (17) | 4 (24) | 10 (59) | 14 (82) | 3 (18) |

[a]For explanation of treatments, see Table I. [b]Significantly different from the value for Group 2 at $P < 0.05$.

**Table III** Serum gastrin and labelling indices of colonic mucosa and cancers and apoptotic index of colon cancer in AOM-treated rats

| Experimental week | Group no. | Treatment[a] | Serum gastrin (pg $ml^{-1}$) | Labelling index: Distal portion | Proximal portion | Colon cancer | Apoptotic index of colon cancer (%) |
|---|---|---|---|---|---|---|---|
| 9 | 1 | Neurotensin | 315 ± 45 | 0.31 ± 0.02 | 0.32 ± 0.02 | | |
| | 2 | Olive oil | 311 ± 39 | 0.31 ± 0.01 | 0.32 ± 0.02 | | |
| 40 | 1 | Neurotensin | 289 ± 28 | 0.24 ± 0.02 | 0.28 ± 0.03 | 0.41 ± 0.03[b] | 2.0 ± 0.2[b] |
| | 2 | Olive oil | 303 ± 43 | 0.24 ± 0.02 | 0.28 ± 0.07 | 0.25 ± 0.02 | 3.3 ± 0.2 |

[a]For explanation of treatments, see Table I. [b]Significantly different from the value for Group 2 at $P < 0.001$.

colon. However, it significantly increased the labelling indices of colon cancers. These findings indicate that enhancement by neurotensin of colon carcinogenesis may be related to its effect of increasing proliferation of colon cancer cells.

Although the mechanism(s) of this effect of neurotensin is unknown, three possible explanations can be considered. The first is the trophic action of gastrin. McGregor *et al.* (1982) induced colon cancer in rats with methylazoxymethanol, and found that chronic exposure to elevated serum gastrin levels induced by antral exclusion augmented the size but not the number of developing tumours. Svet-Moldavsky (1980) reported that gastrin stimulated growth of transplanted adenocarcinomas derived from colon, whereas growth of other tumours, such as stomach sarcoma, hepatoma, sarcoma of the rectum, and adenocarcinoma of the small intestinal tract, was not affected. However, in the present work we found that administration of neurotensin had no influence on the serum gastrin level. A second possible explanation is the effect of neurotensin on intracellular guanosine 3':5'-cyclic monophosphate (cGMP). DeRubertis *et al.* (1976) found that the cGMP content of human colon adenocarcinomas was greater than that of the surrounding mucosa. Neurotensin can increase intracellular cGMP concentration in some cell lines (Amar *et al.*, 1985). The third possibility is the effect of neurotensin on activation of phosphatidylinositol turnover. Amar *et al.* (1986) found that neurotensin had little effect on cyclic nucleotide levels in the human colon adenocarcinoma cell line HT29, but that it strongly stimulated phosphatidylinositol turnover. These results indicate that neurotensin may regulate intracellular $Ca^{++}$ levels in HT29 cells by using inositol triphosphate as a second messenger.

Our results show that neurotensin enhances the growth of colon tumours. Although the exact mechanism(s) of this effect requires further investigation, it may be related to the enhancement by neurotensin of proliferation of colon cancer cells.

### References

AMAR, S., MAZELLA, J., CHECLER, F., KITABCHI, P. & VINCENT, J.-P. (1985). Regulation of cyclic GMP levels by neurotensin in neuroblastoma clone N1E-115. *Biochem. Biophys. Res. Commun.*, **129**, 117.

AMAR, S., KITABCHI, P. & VINCENT, J.-P. (1986). Activation of phosphatidylinositol turnover by neurotensin receptors in the human-colonic adenocarcinoma cell line HT29. *FEBS Lett.*, **201**, 31.

CARRAWAY, R., KITABCHI, P. & LEEMAN, S. (1978). The amino acid sequence of radioimmunoassayable neurotensin from bovine intestine: identity to neurotensin from hypothalamus. *J. Biol. Chem.*, **253**, 7996.

CARRAWAY, R. & LEEMAN, S. (1978). The amino acid sequence of a hypothalamic peptide, neurotensin. *J. Biol. Chem.*, **250**, 1907.

DERUBERTIS, F.R., CHAYOTH, R. & FIELD, J.B. (1976). The content and metabolism of cyclic adenosine 3',5'-monophosphate and cyclic guanosine 3'-5'-monophosphate in adenocarcinoma of the human colon. *J. Clin. Invest.*, **57**, 641.

EASTWOOD, G.L. & QUIMBY, G. (1983). Effect of chronic cimetidine ingestion of fundic and antral epithelial proliferation in the rat. *Dig. Dis. Sci.*, **28**, 61.

FEURLE, G.E., MÜLLER, B., OHNHEISER, G. & BACA, I. (1985). Action of neurotensin on size, composition, and growth of pancreas and stomach in the rat. *Regulat. Peptides.*, **13**, 53.

FEURLE, G.E., MÜLLER, B. & RIX, E. (1987). Neurotensin induces hyperplasia of the pancreas and growth of the gastric antrum in rats. *Gut*, **28**, 19.

GRATZNER, H.G. (1982). Monoclonal antibody to 5-bromo- and 5-iododeoxyuridine: a new reagent for detection of DNA replication. *Science*, **218**, 474.

IINUMA, K., IKEDA, I., TAKAI, M., YANAGAWA, Y. & KURATA, K. (1982). Gastrin radioimmunoassay with polyethylene glycol method. *Radiosotopes*, **31**, 350.

KERR, J.F.R. & SEARLE, J. (1973). Deletion of cells by apoptosis during castration-induced involution of the rat prostate. *Virchows Arch. B. Cell Pathol.*, **13**, 87.

MCGREGOR, D.E., JONES, R.D., KARLIN, D.A. & ROMSDAHL, M.M. (1982). Trophic effects of gastrin on colorectal neoplasms in the rat. *Ann. Surg.*, **195**, 219.

MILLER, R.G. Jr (1966). *Simultaneous Statistics Inference*. McGraw-Hill: New York.

MORSTYN, G., HSU, S.M., KINSELLA, T., GRATZNER, H., RUSSO, A. & MITCHELL, J.B. (1983). Bromodeoxyuridine in tumors and chromosomes detected with monoclonal antibody. *J. Clin. Invest.*, **72**, 1844.

POLAK, J., SULLIVAN, S., BLOOM, S.R. & 4 others (1977). Specific localisation of neurotensin to the N cell in human intestine by radioimmunoassay and immunocytochemistry. *Nature*, **270**, 183.

SIEGEL, S. (1956). *Non-parametric Statistics for the Behavioral Sciences*. McGraw-Hill: New York.

SNEDECOR, C.W. & COCHRAN, W.G. (1967). *Statistical Methods*. Iowa University Press: Ames IA.

STEFANINI, M., DEMARTINE, C. & ZAMBONI, L. (1967). Fixation of ejaculated spermatozoa for electron microscopy. *Nature*, **216**, 173.

SUNTER, J.P., APPLETON, D.R., WRIGHT, N.A. & WATSON, A.J. (1978). Pathological features of the colonic tumours induced in rats by the administration of 1,2-dimethylhydrazine. *Virchows Arch. B. Cell Pathol.*, **29**, 211.

SVET-MOLDAVSKY, G.J. (1980). Dependence of gastro-intestinal tumors on gastro-intestinal hormones: pentagastrin stimulates growth of transplanted colon adenocarcinoma in mice. *Biomedicine*, **33**, 249.

TADA, T., KODAMA, T., WATANABE, S., SATO, Y. & SHIMOSATO, T. (1985). Cell kinetics studies by the use of anti-bromodeoxyuridine monoclonal antibody and their clinical application. *Igaku-no-ayumi*, **135**, 510.

TATSUTA, M., IISHI, H., BABA, M. & TANIGUCHI, H. (1989). Promotion by neurotensin of gastric carcinogenesis induced by N-methyl-N'-nitro-N-nitrosoguanidine. *Cancer Res.*, **49**, 843.

TATSUTA, M., ITOH, T., OKUDA, S., TAMURA, H. & YAMAMURA, H. (1977). Effect of fundusectomy on serum and antral gastrin levels in rats. *Gastroenterology*, **27**, 78.

TERADA, N., YAMAMOTO, R., TAKADA, T. & 6 others (1989). Inhibitory effect of progesterone on cell death of mouse uterine epithelium. *J. Steroid Biochem.*, **33**, 1091.

WALSH, J.H. (1987). Gastrointestinal hormones. In: *Physiology of the Gastrointestinal Tract*, Vol. 1, Johnson, L.R. (ed.) p. 181. Raven Press: New York.

WOOD, J.G., HOANG, H.D., BUSSJAEGER, L.J. & SOLOMON, F.E. (1988). Neurotensin stimulates growth of small intestine in rats. *Am. J. Physiol.*, **255**, G813.